# A Study of Nuclear Transcription Factor-Kappa B in Childhood Autism

**DOI:** 10.1371/journal.pone.0019488

**Published:** 2011-05-09

**Authors:** Usha S. Naik, Charitha Gangadharan, Kanakalatha Abbagani, Balakrishna Nagalla, Niranjan Dasari, Sunil K. Manna

**Affiliations:** 1 Department of Psychiatry, Osmania Medical College, Hyderabad, India; 2 Laboratory of Immunology, Centre for DNA Fingerprinting and Diagnostics, Nampally, Hyderabad, India; 3 National Institute of Nutrition, Hyderabad, India; University of Michigan, United States of America

## Abstract

**Background:**

Several children with autism show regression in language and social development while maintaining normal motor milestones. A clear period of normal development followed by regression and subsequent improvement with treatment, suggests a multifactorial etiology. The role of inflammation in autism is now a major area of study. Viral and bacterial infections, hypoxia, or medication could affect both foetus and infant. These stressors could upregulate transcription factors like nuclear factor kappa B (NF-κB), a master switch for many genes including some implicated in autism like tumor necrosis factor (TNF). On this *hypothesis*, it was proposed to determine NF-κB in children with autism.

**Methods:**

Peripheral blood samples of 67 children with autism and 29 control children were evaluated for NF-**κ**B using electrophoretic mobility shift assay (EMSA). A phosphor imaging technique was used to quantify values. The fold increase over the control sample was calculated and statistical analysis was carried out using SPSS 15.

**Results:**

We have noted significant increase in NF-κB DNA binding activity in peripheral blood samples of children with autism. When the fold increase of NF-κB in cases (n = 67) was compared with that of controls (n = 29), there was a significant difference (3.14 vs. 1.40, respectively; *p*<0.02).

**Conclusion:**

This finding has immense value in understanding many of the known biochemical changes reported in autism. As NF-κB is a response to stressors of several kinds and a master switch for many genes, autism may then arise at least in part from an NF-κB pathway gone awry.

## Introduction

Autism is a severe developmental disorder of childhood. Children with autism demonstrate deficits in social interaction, verbal and nonverbal communication, and repetitive behaviors or interests. Incidence figures range from 4 per 10,000 in 1984 to 16.8 per 10,000 in 2001 [1]. The condition, first described by Leo Kanner (1943), is still diagnosed on the basis of symptoms using the Diagnostic and Statistical Manual 4^th^ edition (DSM IV) Criteria for Autism [2], [3]. Generally 20–25% of cases are considered to be of the regressive type, wherein parents report a period of normal or near normal development followed by developmental plateauing or regression [1]. Lord et al. (2004) reported that 40% of children with autism have regressive autism [4]. The etiology of autism continues to be debated.

Inflammation is now one of the major areas of study in autism. Licinio in an Editorial in ‘Molecular Psychiatry’ suggested that in future there will be a sub category of ‘Autoimmune autism’ within the autism spectrum [5]. Jyonouchi and others (2001) studied proinflammatory and regulatory cytokines to understand innate and adaptive immune responses in children with autism who had developmental regression [6]. Peripheral blood mononuclear cells (PBMCs) were stimulated with lipopolysaccharide (LPS) in three groups of children – Children with Autism Spectrum Disorder (ASD), normal siblings, and controls. The ASD group produced significantly more proinflammatory cytokines namely, tumor necrosis factor α (TNFαand interleukins, especially IL-1ß and IL-6. In some children the ratio of proinflammatory/counter-inflammatory cytokines, TNFα/TNFRII (tumor necrosis factor receptor II) was also higher. This could imply aberrant innate responses. IL-10 production was lower in PBMCs stimulated with IL-18. This suggests a dysregulation of the adaptive immune mechanism as well [6], [7]. An important recent review “The immune system's role in the biology of autism” has highlighted the role of immune system dysfunction in autism. Among the cytokines studied, levels of transforming growth factor beta (TGFβ) were reduced in plasma, while levels of macrophage inhibitory factor (MIF) were increased. Lower levels of immunoglobulins, differences in gene expression related to natural killer cell activity and altered monocyte cytokine responses to Toll-like receptor (TLR) stimulation, have all been documented in ASD. Much work has been done on circulating maternal antibodies directed towards brain proteins [8].

**Figure 1:**
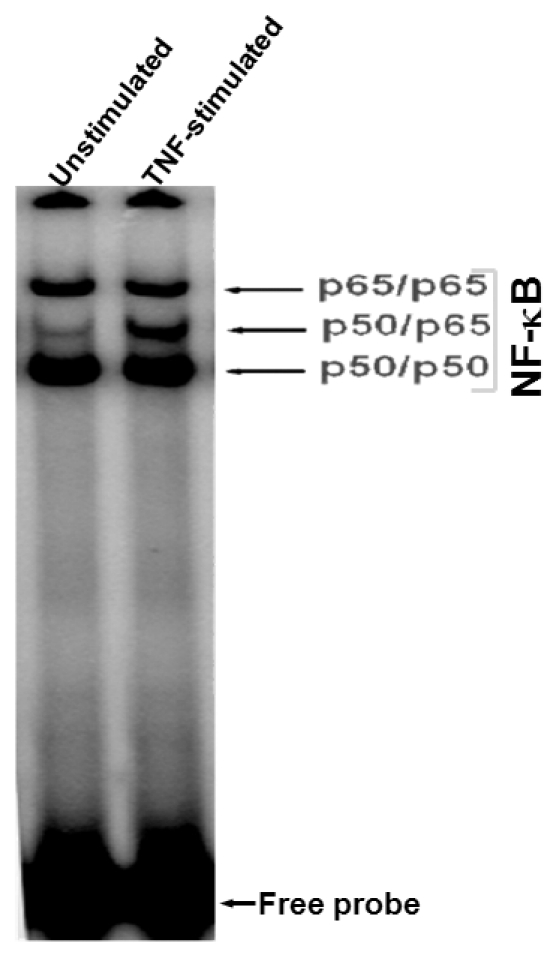
Composition of NF-κB band. Nuclear extracts from untreated and TNF-stimulated cells were detected by gel shift assay and showed different dimers of NF-κB.

NF-κB is an important regulator of immune mechanisms and alterations in its activity could explain many of these observations. David Baltimore, in whose laboratory NF-κB was first discovered, states “In 1986, my laboratory was searching for transcription factors that might control the activation of the kappa immunoglobulin light chain when we came across NF-κB. We thought it was specific to B-lymphocytes and never imagined that it would turn out to be among the most protean of transcription factors ever discovered – ready to be activated by a wide range of inducing stimuli” [9]. Under normal conditions, NF-κB is present in the cytoplasm as an inactive heterotrimer (Figure 1). Stimulation with a specific inducer, such as TNFα, activates an IκB kinase complex (IKKs), triggering its degradation and allowing free NF-κB to translocate to the nucleus and activate gene expression [10]–[12]. NF-κB activity is needed for proper immune system function while constitutive activation of NF-κB pathway is associated with malignancies and various inflammatory diseases [11], [13]. This transcription factor in turn is a master switch for many genes, including some implicated in autism like tumor necrosis factor (TNF) [11]–[13]. Moreover, oxidative stress leads to increase in reactive oxygen species (ROS) which activates IKKs that phosphorylate IκBα for proteasomal degradation [14]. When ROS is inhibited, the activation of NF-κB is abrogated [15], [16]. Oxidative stress has been shown to contribute to the etio pathology of various disorders including autism. In a recent study, cerebellar levels of 3-nitrotyrosine, a known marker for oxidative stress, were found to be significantly increased in autism [17]. Chez et al reported that TNFα,a potent inducer of NF-κB, was significantly higher in the cerebrospinal fluid of children with autism [18].

Children diagnosed with autism, have an over representation of birth by Cesarean Section, neonatal nursery admissions, repeated infections and repeated antibiotic use [19]. The impact of early life programming and changes in transcription factors is an emerging area of interest in neurodevelopment [20]. As several of these factors represent different kinds of stressful events, which could up regulate NF-**κ**B, it was proposed to study this transcription factor. With informed consent and with clearance from the ethics committee of the Osmania Medical College, it was proposed to study NF-κB DNA binding activity in children with autism and regression, and in a group of control children matched for age. The objectives were to understand the role of NF-κB in childhood autism in 50 children and 30 controls. In this study, we have noted a significant increase in NF-κB DNA binding activity in peripheral blood samples of children with autism. When fold increase of NF-**κ**B in children with autism was compared with that of age matched controls, there was a significant difference between the two groups.

## Results

**Figure 2:**
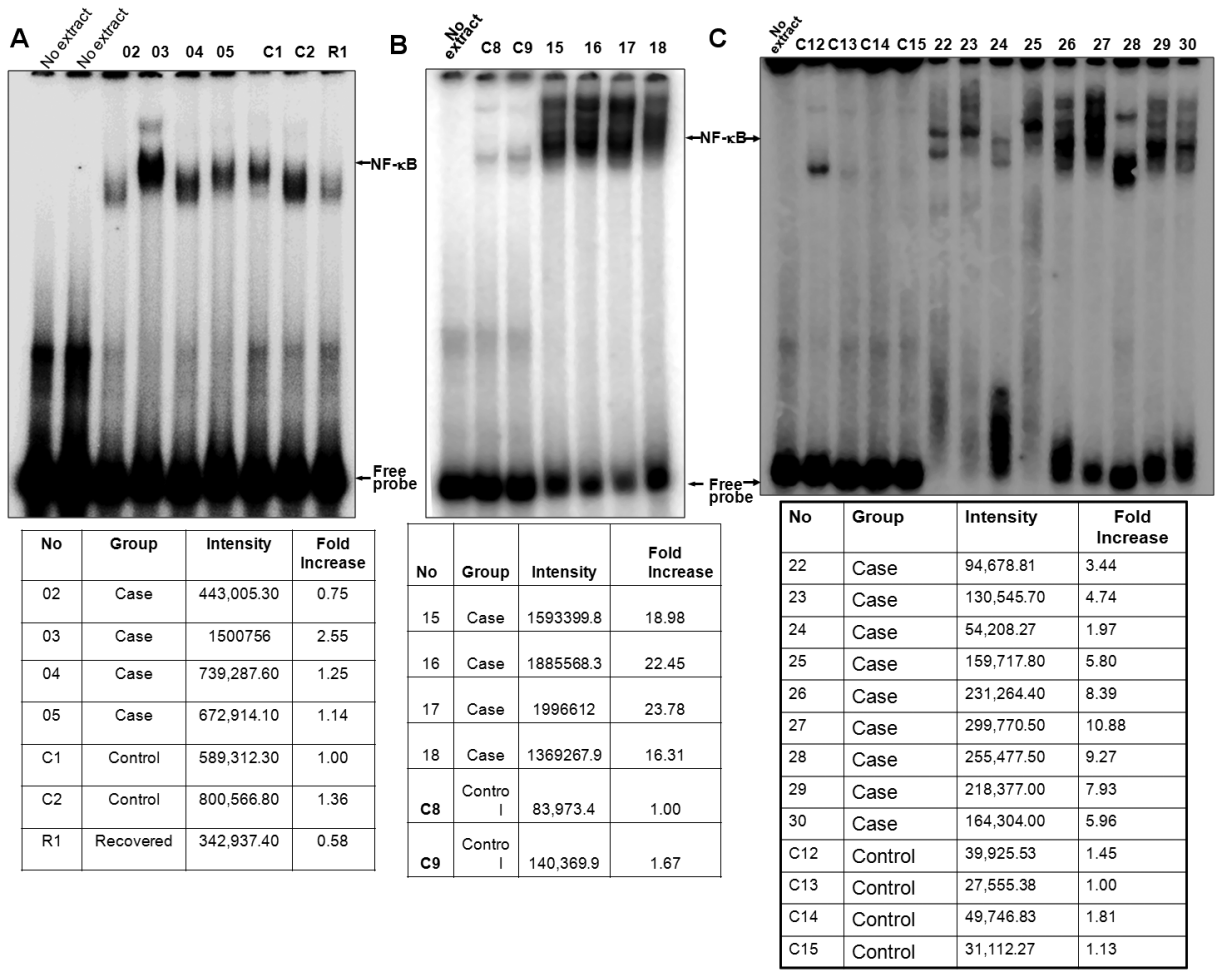
Amount of NF-κB DNA binding of cases, controls, and recovered (R1) children. PBMC were separated from fresh peripheral blood of children with autism and age matched controls by 2.5% gelatin sedimentation followed by Ficoll-paque density gradient centrifugation. The pellet was used to prepare nuclear and cytoplasmic extracts. 8 µg of nuclear extract was assayed for NF-κB DNA binding using EMSA. Radioactive bands were detected from dried gel after exposure in the Phosphorscreen. The amounts of NF-κB DNA binding (A, left upper panel) and the fold increase (A, left lower panel) of 4 cases (02–05), 2 controls (C1 and C2) and 1 recovered (R1) were indicated. The amounts of NF-κB DNA binding (B, right upper panel) and the fold increase (B, right lower panel) of 4 cases (15–18) and 2 controls (C8 and C9) were indicated. Similarly, the amounts of NF-κB DNA binding (C, left panel) and the fold increase (C, right panel) of 9 cases (22–30) and 4 controls (C12–C15) were indicated.

**Figure 3:**
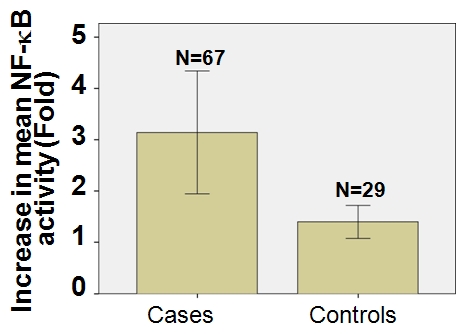
The mean fold intensities of NF-κB DNA binding in cases and controls. 67 cases (mean fold intensity is 3.1408) and 29 age matched controls (mean fold intensity is 1.398) *p*<0.02 is depicted.

The number of cases on which NF-κB detection (both DNA binding and fold of activation of DNA binding) was completed with controls for comparison was 67 children (Fig. 2). The overall fold activation of NF-κB is depicted in Figure 3.

As shown in Table 1 the total number of cases with autism was 67. There were 29 age matched controls and 2 adult controls. Three children had recovered symptom wise from autism and were no longer autistic on the Childhood Autism Rating Scale (CARS) and were tested to compare values of markers [21].

**Table 1:** Frequency distribution, age and gender.

	Number	Mean Age	Males	Females
**Cases**	67	4.1424	64	3
**Controls**	29	4.0513	19	8
**‘Recovered Cases’**	3	7.0	3	-
**Adult Controls**	2	35	-	2
**Total**	101			

Of the cases, there were 64 males and 3 females. The usual ratio reported is 5–6 males per affected female. The controls were more equally distributed. The mean ages ranged from 18 months to ten years with 4.14 years for cases and 4.05 years for controls.

Four sets of data were analyzed -

- Using the data from the current study with 67 cases and 29 age matched controls.
- 67 cases and 29 age matched controls plus two adult controls - a total of 31 controls.
- Data, excluding four cases which caused skew by exceptionally high values - 63 cases and 31 controls (Fig. 2B).
- Three children, those no longer tested as autistic on CARS, were evaluated against the autistic group. Details are provided in Text S1. Two children had completed five years of treatment each and one child was on treatment for three years when the samples were collected.

The mean fold intensities were 3.1408 for cases and 1.3980 for controls (Table 2). This is also depicted in Figure 3. The mean fold increase was statistically significant between cases and controls (*p*<0.02) using the Mann Whitney U Test (Table 2). The variances of fold increase were significantly different among the groups therefore the Mann Whitney U Test was used to compare mean values. The mean rank for cases was 52.85 and the mean rank for controls was 38.45. This difference was significant (*p*<0.02).

**Table 2:** Mean fold intensity of NF-κB DNA binding in children with autism (cases) vs. without autism (controls).

	Groups	Cases	Controls	*p* value
A	67 cases and 29 age matched, controls	3.1408	1.3980	0.02
B	67 cases and 31 controls(2 Adults)	3.1408	1.3723	0.01
C	Data excluding four cases causing a skew in data by exceptionally high values63 cases and 31 controls	2.0464	1.3723	0.028
D	Three recovered' cases and 67 casesWhen tested on CARS	Cases	Recovered	
		3.1408	0.7741	0.026

Comparison of mean fold intensities between groups by Non parametric test of ‘Mann-Whitney U’ was used.

Childhood Autism Rating Scale (CARS) [21].

In all the four sets of data, statistical analysis was significant. A larger sample in group D would be very gratifying to work with. The details of statistical analysis of groups B, C, and D are in the Text S1.

## Discussion

Nuclear transcription factor kappaB is a stress inducible transcription factor. We have provided data to support the role of NF-κB in autism. No control study has been reported on NF-κB in children with autism to the best of our knowledge. In our study, we have found that there was a significant increase in the amount of NF-κB in samples from children with autism when compared with those from age matched controls. Studies are beginning to document that recovery or “optimal development” bringing the child out of the autism spectrum, is possible [22], [23]. Our finding in three recovered children needs further evaluation in this context.

Elevated amounts of NF-κB in children with autism can strengthen the conceptual frameworks of the role of innate immunity and ROS in the etiopathology of this condition. A new study CHARGE (Childhood Autism Risks from Genetics and Environment) has found a significant increase in a number of cytokines in plasma including IL-1β, IL-6, IL-8, and IL-12 p40 in an ASD group compared with typically developing controls [24]. All of these cytokines are known to be regulated by NF-κB [11]. Using a proteomic approach Shen et al. (2010) found that in a multiplex family with autism, the amount of p52 was increased without change in the amount of IκBα and postulated that NF-κB could be activated in a non-canonical manner [25]. However, the authors did not evaluate the activity of NF-κB in the nucleus. The most important study of inflammatory markers in autism comes from an autopsy series [26], [27]. Macrophage chemoattractant protein (MCP-1) and tumor growth factor beta-1 (TGFβ-1), known neuroinflammatory markers, were found to be consistently increased [26], [27]. For example, the ligand for Fms-related tyrosine kinase 3 (Flt3), a VEGF receptor, known to promote the proliferation and mobilization of hematopoietic cells is an NF-κB-dependent gene and elevated levels were found in brain tissue of patients in this study [26], [28]. Since NF-κB is known to be a major regulator of innate immunity and these cytokines have been found to be up regulated by NF-κB, our finding of significant elevation of this transcription factor becomes even more relevant.

An important mechanism of activation of NF-κB is through the production of ROS. ROS generation is related to stress which could be due to multiple environmental, behavioral, and concomitant illness factors [29]. Impaired methylation and increased oxidative stress have been found in children with autism [30]. More recently, the oxidative stress marker 3-nitrotyrosine (3-NT) was reported to be significantly elevated in cerebellar tissue homogenates of individuals with autism when compared to normal controls [17]. The presence of markers of oxidative stress, known to modulate NF-κB, in brain tissue of individuals with autism is an important correlation. Children with autism could be in a “hyper arousal” state of NF-κB due to the constant effect of environmental stressors – even fear is known to upregulate NF-κB [11]. Children with autism may have an altered threshold to fearful stimuli.

Recently, the mechanisms underlying the termination of NF-κB activity have been discussed [31], [32]. Children with autism may be unable to turn off stress induced responses. Terminating NF-κB activity is dependent on any of several downstream modulators. These operate variously through altered cofactor binding, degradation and displacement of NF-κB from DNA. These modulators are worth studying like the suppressor of cytokine signaling 1 (SOCS1) and several inhibitors of the IκB family. TNFα has been shown to be in excess in the serum and CSF of individuals with autism [33]. It is known to be a potent inducer of NF-κB and is also in turn unregulated by NF-κB. Azadirachtin, derived from neem, has recently been shown to block TNF-induced biological responses by inhibiting ligand binding [34]. Drugs like this could be of potential use in autism. Conversely, identifying agents that increase NF-κB in children and regulating these triggers, would go a long way in preventing a certain sub sect of regressive autism.

NF-κB has rightly been called a double edged sword, both needed by the body in its defense and producing disease when inappropriately activated. To conclude, several neurological and inflammatory disorders have been linked to NF-κB. Autism, our results tell us, now appears to have joined their ranks.

## Materials and Methods

### Clearance from the Ethics committee of the Osmania Medical College

Clearance was taken from the ethics Committee of the Medical College before commencement of the study, to draw blood samples (3 ml from each child), of children with autism and from control children.

### Selection of cases

Cases attending the Department of Child Psychiatry, Niloufer Hospital were screened for features of autism. Each child was assessed by the same team, comprising a psychiatrist, clinical psychologist and psychiatric social worker. Children who fulfilled DSM IV criteria were further assessed for regression. Regression was defined, here as a loss of previously achieved milestones in language, personal or social development or in adaptive skills. Children with regression in motor milestones were excluded. All children presented to us after several months of regression. The manner in which regression was assessed is detailed in Text S2.

Children were selected as cases, if

- they fulfilled DSM IV criteria,
- had normal motor milestones, but had regressed in at least one other area of development,
- were aged between 18 months and 10 years,
- parents were ready to participate in the study, and ready to sign the consent form.

### Selection of controls *

Children were selected as controls if –

- they were age matched,
- from the same family,
- did not show other abnormalities on a questionnaire to exclude psychiatric disorders,
- had normal motor milestones,
- were aged between 18 months and 10 years,
- parents were ready to participate in the study, and ready to sign the consent form

* One of the problems we faced was that most children were single and while there was no shortage of referred cases, it was difficult obtaining controls. All controls were either siblings or cousins of the affected child. The children were matched for age and socioeconomic status but not for gender. The Consent form is detailed in Text S3. There were 29 age matched controls and 2 *adult* controls. The adults were taken as controls, on two occasions, when scheduled control children did not show up and the samples of children with autism would otherwise have been wasted.

### Sample collection

3 ml blood was drawn in Na-EDTA vials and used for experiments.

### Test procedures

#### Isolation of PBMC and neutrophils from human blood

Neutrophils and peripheral blood mononuclear cells (PBMC) were separated from fresh peripheral blood by 2.5% gelatin sedimentation followed by ficoll-paque (Histopaque-1077) density gradient centrifugation method. The EDTA-blood was incubated with 2.5% gelatin (in saline) solution in saline with 1∶1 ratio at 37°C for 30 minutes. The erythrocytes (RBC) were sedimented in 30 minutes as these cells formed rouleaux. The RBC-free upper layer (rich with leukocytes and platelets) formed above the Histopaque and was centrifuged at 700×g for 30 min. This layer (PBMC) was carefully removed by aspiration, suspended in phosphate buffer saline (PBS) and centrifuged at 1500×g for 5 minutes. The pellet was washed twice with PBS and then used to prepare cytoplasmic and nuclear extracts.

#### Electrophoretic mobility shift assay (EMSA)

PBMC were assayed for NF-κB activation using EMSA (electrophoretic mobility shift assay). Cells were suspended in 0.4 ml of lysis buffer (10 mM HEPES (pH 7.9), 10 mM KCl, 0.1 mM EDTA, 0.1 mM EGTA, 1 mM DTT, 0.5 mM PMSF, 2 µg/ml leupeptin and 2 µg/ml aprotinin and incubated on ice for 15 min, after which 12.5 µl of 10% Nonidet P-40 was added. The tube was then vigorously shaken on a vortex machine for 10 s, and the homogenate centrifuged at maximum speed for 30 s in a microfuge. The nuclear pellet was re-suspended in 25 µl of ice-cold nuclear extraction buffer (20 mM HEPES (pH 7.9), 0.4 M NaCl, 1 mM EDTA, 1 mM EGTA, 1 mM DTT, 1 mM PMSF, 2.0 µg/ml leupeptin and 2.0 µg/ml aprotinin). The tube was incubated on ice for 30 min with intermittent mixing and finally centrifuged for 5 min in a microfuge at 4°C, and the supernatant (nuclear extract) collected. 8 µg of nuclear extract was incubated in a mixture containing 2 µg of poly dI∶dC in a binding buffer (25 mM HEPES pH 7.9, 0.5 mM EDTA, 0.5 mM DTT, 1% Nonidet P-40, 5% glycerol, and 50 mM NaCl) with double-stranded oligonucleotide of NF-κB. EMSA was performed in native gel. The gel was dried and exposed in a phosphor screen and scanned in a Phosphor Imager (Fuji Photo Film, Japan).

### Quantification of intensity

The software used for quantification was Image Quant 5.2. To quantify the intensity, an equal area was selected from each lane and the value from the free probe lane (without any sample/negative control/only the radioactive master mix) was subtracted from the test lanes. To deduce the fold intensity, the lowest control value was taken as 1 fold. The patient values were divided by the control values to obtain fold values. Some of the controls also had high values. However, all control values were included in calculation of the mean for the control group and in statistical analysis.

### Statistical Analyses

Data was analyzed using SPSS Version 15. Mean and SD values were calculated for fold increase by groups, as shown in Figure 2. The complete data are included in Text S4. The mean values of fold increase among groups were compared using student t- test and the Mann Whitney U Test as the variance between groups was high.

Statistical tests used were both parametric and non parametric as data was skewed.

## Supporting Information

Text S1 Details of statistical analysis of groups.
(DOC)

Text S2 Evaluation of regression.
(DOC)

Text S3 Consent forms.
(DOC)

Text S4 Fold intensity of cases and controls for NF-κB DNA binding.
(DOC)
