# Supplementary material for: A Study of Nuclear Transcription Factor-Kappa B in Childhood Autism
Source: PLoS One. 2011 May 9;6(5):e19488. doi: 10.1371/journal.pone.0019488 (PMC3090385; doi:10.1371/journal.pone.0019488)
Supplement: Text S1 — Details of statistical analysis of groups. (DOC) [file pone.0019488.s001.doc]

**Text S1: Details of Statistical analysis of groups.**

Statistical analysis was carried out using 67 cases and 29 age matched controls plus two adult controls (31 controls).

|  | **N** | **Mean** | **Std. Deviation** |
| --- | --- | --- | --- |
| Cases | 67 | 3.1408 | 4.91035 |
| Controls | 31 | 1.3723 | .82206 |
| Total | 98 | 2.5814 | 4.15910 |

## Table A.1: Mean fold intensities by groups.

| **Levene**  **Statistic** | **df1** | **df2** | **Sig.** |
| --- | --- | --- | --- |
| 12.667 | 1 | 96 | .001 |

*p*<0.001

## Table A.2: Test of Homogeneity of Variances.

|  | **Group** | **N** | **Mean**  **Rank** | **Sum of**  **Ranks** |
| --- | --- | --- | --- | --- |
| **FOLD**  **Increase** | Cases | 67 | 54.49 | 3651.00 |
|  | Controls | 31 | 38.71 | 1200.00 |
|  | Total | 98 |  |  |

Table A.3: Mann-Whitney U Test and Ranks.

|  | **FOLD Intensity** |
| --- | --- |
| Mann-  Whitney U  704.000  Asymp. Sig.  (2-tailed) | 704.000  0.010 |

Table A.4: Mann-Whitney U statistic and Level of Significance.

**B) Data excluding four case causing a skew in data by exceptionally high values 63 cases and 31 controls.**

|  | **N** | **Mean** | **Std.**  **Deviation** |
| --- | --- | --- | --- |
| Cases | 63 | 2.0464 | 2.17390 |
| Controls | 31 | 1.3723 | .82206 |
| Total | 94 | 1.8241 | 1.86281 |

Table B.1: Mean fold intensities by groups.

| **Levene**  **Statistic** | **df1** | **df2** | **Sig.** |
| --- | --- | --- | --- |
| 7.754 | 1 | 92 | .007 |

Table B.2: Test of Homogeneity of Variances (Fold intensities).

There was a difference in mean rank values between cases and controls (Table B.3) with statistical significance (Table B.4) even when 4 cases with very high values were removed.

|  | **Group** | **N** | **Mean**  **Rank** | **Sum of**  **Ranks** |
| --- | --- | --- | --- | --- |
| **FOLD**  **Intensity** | Cases | 63 | 51.83 | 3265.00 |
|  | Controls | 31 | 38.71 | 1200.00 |
|  | Total | 94 |  |  |

Table B.3: Mann-Whitney U Test and Ranks.

|  | **FOLD Intensity** |
| --- | --- |
| Mann-  Whitney U  704.000  Asymp. Sig.  (2-tailed) | 704.000  .028 |

Table B.4: Mann-Whitney U statistic and Level of Significance.

**C) Three ‘recovered ‘cases.**

|  | **N** | **Mean** | **Std.**  **Deviation** |
| --- | --- | --- | --- |
| Cases | 67 | 3.1408 | 4.91035 |
| Recovery | 3 | .7741 | .24718 |
| Total | 70 | 3.0394 | 4.82681 |

Table C.1: Mean fold intensities by groups.

| **Levene**  **Statistic** | **df1** | **df2** | **Sig.** |
| --- | --- | --- | --- |
| 1.550 | 1 | 68 | .217 |

Table C.2: Test of Homogeneity of Variances FOLD Intensity.

|  | **N** | **Mean**  **Rank** | **Sum of**  **Ranks** |
| --- | --- | --- | --- |
| Cases | 67 | 36.61 | 2453.00 |
| Recovery | 3 | 10.67 | 32.00 |
| Total | 70 |  |  |

Table C.3: Mann-Whitney U Test and Ranks.

|  | **FOLD Intensity**  **Increase** |
| --- | --- |
| Mann-Whitney U 26.000  Asymp. Sig. (2-  tailed)  .031  Exact Sig. [2*(1-  tailed Sig.)] | 26.000  .031  .026(a) |

Table C.4: Mann-Whitney U statistic and Level of Significance.

Though the numbers are small, the difference between cases and ‘Recovered’ children is interesting (p<0.026).
