# Supplementary material for: A Study of Nuclear Transcription Factor-Kappa B in Childhood Autism
Source: PLoS One. 2011 May 9;6(5):e19488. doi: 10.1371/journal.pone.0019488 (PMC3090385; doi:10.1371/journal.pone.0019488)
Supplement: Text S2 — Evaluation of regression. (DOC) [file pone.0019488.s002.doc]

**Text S2: Evaluation of Regression.**

1. **A preliminary screening for important milestones**

**Assessment for Regression –A**

1. **A Birthday related approach**

**Assessment for Regression -B**

1. **A detailed evaluation of Regression**

**Assessment for Regression-C**

**I. A preliminary screening for important milestones.**

**Developmental Milestones:** Age of attaining important Milestones, particularly Birth to two years. Particular attention should be paid to details as they can prove very informative in understanding pathways of development like, milestone lost, milestone lost and regained with meaning /without meaning.

Language and Social Milestones need to be reviewed with the following questions:

Attained: example - Mama, Daddy Specific with meaning.

Attained and continued: example - Mama, Daddy with meaning.

Lost and regained with/ without meaning: example - says Mama, Daddy without reference to parent.

**I. A preliminary screening for important milestones Assessment for Regression (A)**

| **Milestone** | **Age**  **Attained** | **Continues**  **Same** | **Continues**  **Meaning**  **Lost** | **Lost** | **Age**  **Lost** | **Lost &**  **Regained** |
| --- | --- | --- | --- | --- | --- | --- |
| Social Smile |  |  |  |  |  |  |
| Neck Holding |  |  |  |  |  |  |
| Stranger Anxiety |  |  |  |  |  |  |
| Sitting without support |  |  |  |  |  |  |
| Poly syllabalic babbling |  |  |  |  |  |  |
| Turns to name |  |  |  |  |  |  |
| Walks without support |  |  |  |  |  |  |
| Two words with meaning |  |  |  |  |  |  |
| Waves Bye |  |  |  |  |  |  |
| Indicates toilet needs |  |  |  |  |  |  |
| Puts two words together |  |  |  |  |  |  |
| Indicates affection on  Reuniting with parents  (e.g. father after work) |  |  |  |  |  |  |

**II. A Birthday related approach Assessment for Regression (B)**

Two specific questions seem relevant-How did the child appear at the first birthday.

How did the child appear at the second birthday? Third birthday was included, because of the definition “Onset before three years”-DSM IV Criteria”

| **No.** | **Milestone** | **First**  **Birthday** | **Second**  **Birthday** | **Third**  **Birthday** |
| --- | --- | --- | --- | --- |
| 1 | Normal Development |  |  |  |
| 2 | Change or delay noticed in Language |  |  |  |
| 3 | Change or delay noticed in Motor Development |  |  |  |
| 4 | Change or delay noticed in Fine motor Development |  |  |  |
| 5 | Change or delay noticed in Personal Development |  |  |  |
| 6 | Change or delay noticed in Social Development |  |  |  |
| 7 | Oddities Apparent |  |  |  |
| 8 | Reduced responsiveness |  |  |  |
| 9 | Did you ever feel the child could not hear? |  |  |  |
| 10 | Did you ever feel the child could not see? |  |  |  |
| 11 | Other concerns Specify |  |  |  |

|  | Nil | <12  mths | 12-18  mths | 18-24  mths | >24  mths | Initial  Delay | Achievement  before  Regression | Plateauing | Anything  unusual  or exceptional  Comments |
| --- | --- | --- | --- | --- | --- | --- | --- | --- | --- |
| Regression in receptive  Language  Turning to voice/sound or Diminished number of responses |  |  |  |  |  |  |  |  |  |
| Regression in receptive Language  Obeying commands |  |  |  |  |  |  |  |  |  |
| Regression in pointing |  |  |  |  |  |  |  |  |  |
| Regression or change in  waving Bye |  |  |  |  |  |  |  |  |  |
| Regression in expressive. Language – speech |  |  |  |  |  |  |  |  |  |
| Regression in bladder control  or indication |  |  |  |  |  |  |  |  |  |
| Regression in bowel control or indication |  |  |  |  |  |  |  |  |  |
| Regression in other  Self help skills |  |  |  |  |  |  |  |  |  |
| Regression in play |  |  |  |  |  |  |  |  |  |
| Regression in social  development |  |  |  |  |  |  |  |  |  |
| Regression in interests,  Curiosity or activities |  |  |  |  |  |  |  |  |  |
| Regression in Sensory/Perceptual  Abilities |  |  |  |  |  |  |  |  |  |
| Any other regression  Noticed |  |  |  |  |  |  |  |  |  |

**III. Assessment for Regression Detailed evaluation of Regression (C)**
