# Supplementary material for: A Study of Nuclear Transcription Factor-Kappa B in Childhood Autism
Source: PLoS One. 2011 May 9;6(5):e19488. doi: 10.1371/journal.pone.0019488 (PMC3090385; doi:10.1371/journal.pone.0019488)
Supplement: Text S3 — Consent forms. (DOC) [file pone.0019488.s003.doc]

**Text S3: Consent forms.**

**CONSENT FORM FOR GIVING BLOOD SAMPLES IN CHILDHOOD AUTISM**

I ............................................................................................................ Father / Mother of

......................................................................... (Psy. No........................) consent to give a blood sample of about 3 ml of my child who has been diagnosed to have childhood autism.

I understand that the sample will be used to test for molecular markers and will contribute to the understanding of the condition.

Signed:

Father / Mother:

Witness:

Date:

**CONSENT FORM FOR GIVING BLOOD SAMPLES IN CHILDHOOD AUTISM**

I.......................................................................................................................... Father / Mother of

..................................................................who is a sibling / cousin, of........................................... (Psy No........................) consent to give a blood sample of about 3 ml of my normal child.

I understand that the sample will be used to test for molecular markers. It will not benefit this child in any way but will contribute to the understanding of the condition.

Signed:

Father / Mother:

Witness: Date:
