# Supplementary material for: A Study of Nuclear Transcription Factor-Kappa B in Childhood Autism
Source: PLoS One. 2011 May 9;6(5):e19488. doi: 10.1371/journal.pone.0019488 (PMC3090385; doi:10.1371/journal.pone.0019488)
Supplement: Text S4 — Fold intensity of cases and controls for NF-κB DNA binding. (DOC) [file pone.0019488.s004.doc]

**Text S4: Fold Intensity of Cases and Controls for NF-κB DNA binding.**

| **S.No** | **Gender** | **Intensity** | **Fold Intensity Increase** | **Group** |
| --- | --- | --- | --- | --- |
|  |  |  |  |  |
| 1 | 1 | 443,005.30 | 0.75 | 1 |
| 2 | 1 | 1500756 | 2.55 | 1 |
| 3 | 1 | 739,287.60 | 1.25 | 1 |
| 4 | 1 | 672,914.10 | 1.14 | 1 |
| **1*** | 1 | 589,312.30 | 1.00 | 2 |
| **2** | 1 | 800,566.80 | 1.36 | 2 |
| R1** | 1 | 342,937.40 | 0.58 | 3 |
| 5 | 1 | 266,028.90 | 1.15 | 1 |
| 6 | 1 | 292,785.10 | 1.27 | 1 |
| 7 | 1 | 585,606.30 | 2.54 | 1 |
| 8 | 1 | 254,010.00 | 1.10 | 1 |
| 9 | 1 | 288,763.50 | 1.25 | 1 |
| **3** | 1 | 913,447.90 | 3.96 | 2 |
| **4** | 2 | 230,955.70 | 1.00 | 2 |
| **5** | 1 | 393,296.10 | 1.70 | 2 |
| **6** | 1 | 1057777.106 | 4.58 | 2 |
| 10 | 2 | 304,290.20 | 1.08 | 1 |
| 11 | 1 | 455,036.70 | 1.61 | 1 |
| 12 | 1 | 346,925.40 | 1.23 | 1 |
| 13 | 1 | 144,130.30 | 0.51 | 1 |
| 14 | 1 | 357,942.80 | 1.27 | 1 |
| **7** | 1 | 282,290.80 | 1.00 | 2 |
| 15 | 1 | 1593399.8 | 18.98 | 1 |
| 16 | 1 | 1885568.3 | 22.45 | 1 |
| 17 | 1 | 1996612 | 23.78 | 1 |
| 18 | 1 | 1369267.9 | 16.31 | 1 |
| **8** | 2 | 83,973.40 | 1.00 | 2 |
| **9** | 1 | 140,369.90 | 1.67 | 2 |
| 19 | 1 | 449,238.40 | 2.10 | 1 |
| 20 | 1 | 303,545.20 | 1.42 | 1 |
| 21 | 1 | 536,341.60 | 2.51 | 1 |
| **10** | 1 | 356,748.20 | 1.67 | 2 |
| **11** | 1 | 213,906.40 | 1.00 | 2 |
| 22 | 1 | 94,678.81 | 3.44 | 1 |
| 23 | 1 | 130,545.70 | 4.74 | 1 |

54

| 22 | 1 | 94,678.81 | 3.44 | 1 |
| --- | --- | --- | --- | --- |
| 23 | 1 | 130,545.70 | 4.74 | 1 |
| 24 | 1 | 54,208.27 | 1.97 | 1 |
| 25 | 1 | 159,717.80 | 5.80 | 1 |
| 26 | 1 | 231,264.40 | 8.39 | 1 |
| 27 | 1 | 299,770.50 | 10.88 | 1 |
| 28 | 1 | 255,477.50 | 9.27 | 1 |
| 29 | 1 | 218,377.00 | 7.93 | 1 |
| 30 | 1 | 164,304.00 | 5.96 | 1 |
| **12** | 2 | 39,925.53 | 1.45 | 2 |
| **13** | 2 | 27,555.38 | 1.00 | 2 |
| **14** | 1 | 49,746.83 | 1.81 | 2 |
| **15** | 1 | 31,112.27 | 1.13 | 2 |
| 31 | 2 | 77,648.15 | 1.89 | 1 |
| 32 | 1 | -2,516.45 | -0.06 | 1 |
| 33 | 1 | 63,926.63 | 1.56 | 1 |
| 34 | 1 | 54,464.66 | 1.33 | 1 |
| **16** | 2 | 41,049.68 | 1.00 | 2 |
| 35 | 1 | 145,016.00 | 0.39 | 1 |
| 36 | 1 | 138,859.90 | 0.37 | 1 |
| 37 | 1 | 427,676.40 | 1.14 | 1 |
| 38 | 1 | 259,058.20 | 0.69 | 1 |
| 39 | 1 | 445,446.70 | 1.18 | 1 |
| **17** | 2 | 376,273.60 | 1.00 | 2 |
| **18** | 2 | 419,372.60 | 1.11 | 2 |
| 40 | 1 | 164,875.30 | 0.94 | 1 |
| 41 | 1 | 204,722.70 | 1.17 | 1 |
| 42 | 1 | 193,805.90 | 1.11 | 1 |
| 43 | 1 | 182,794.80 | 1.05 | 1 |
| 44 | 1 | 198,720.80 | 1.14 | 1 |
| **19** | 1 | 174,747.10 | 1.00 | 2 |
| **20** | 1 | 193,175.50 | 1.11 | 2 |
| 45 | 1 | 317,414.30 | 1.02 | 1 |
| 46 | 1 | 449,640.30 | 1.45 | 1 |
| 47 | 1 | 538,981.80 | 1.73 | 1 |
| 48 | 1 | 415,016.60 | 1.34 | 1 |
| 49 | 2 | 300,819.70 | 1.82 | 1 |
| 50 | 1 | 879,008.30 | 1.40 | 1 |
| 51 | 1 | 1437584 | 2.29 | 1 |
| **24** | 2 | 626,782.40 | 1.00 | 2 |
| 52 | 1 | 9,106.88 | 0.52 | 1 |
| 53 | 1 | 16,118.41 | 0.92 | 1 |
| **25** | 1 | 17,575.15 | 1.00 | 2 |
| 54 | 1 | 40,672.00 | 2.31 | 1 |
| 55 | 1 | 13,334.23 | 0.76 | 1 |
| 26 | 1 | 16,667.13 | 1.76 | 1 |
| 56 | 1 | 27,631.18 | 3.00 | 1 |
| 57 | 1 | 10,323.23 | 1.09 | 1 |
| 58 | 1 | 16,499.57 | 1.74 | 1 |
| **26** | 2 | 9,492.25 | 1.00 | 2 |
| 59 | 1 | 16118.41 | 2.03 | 1 |
| 60 | 1 | 17575.15 | 1.06 | 1 |
| R2 | 1 | 9106.8 | 1.05 | 3 |
| **27** | 1 | 13,532.31 | 1.00 | 2 |
| 61 | 1 | 186450.3 | 0.32 | 1 |
| 62 | 1 | 186485.8 | 0.32 | 1 |
| 63 | 1 | 456500.7 | 0.78 | 1 |
| 64 | 1 | 683641.3 | 1.18 | 1 |
| **28** | 1 | 581551.2 | 1.10 | 2 |
| **29** | 1 | 641595.7 | 1.00 | 2 |
| R3 | 1 | 2228587 | 0.69 | 3 |
| 65 | 1 | 4066313 | 1.25 | 1 |
| **30** | 2 | 3242283 | 1.00 | 2 |
| 66 | 1 | 436202.8 | 1.06 | 1 |
| 67 | 1 | 491305 | 1.19 | 1 |
| **31** | 1 | 411403 | 1 | 2 |

Group 1, Cases; Group 2 Controls; Group 3, ‘Recovered’.

* The Serial Nos. of Controls is in bold.

**R1, R2, and R3 are ‘Recovered Cases’.
